# Clinical and Radiographic Evaluation of Diode and Er,Cr:YSGG Lasers as an Alternative to Formocresol and Sodium Hypochlorite for Pulpotomy Techniques in Primary Molars: A Randomized Controlled Clinical Trial

**DOI:** 10.7759/cureus.65902

**Published:** 2024-07-31

**Authors:** Wasan A Fadhil, Arass J Noori

**Affiliations:** 1 Dentistry, University of Sulaimani, Sulaymaniyah, IRQ

**Keywords:** randomized controlled trial, sodium hypochlorite, formocresol, laser, primary teeth, pulpotomy

## Abstract

Background

Pulpotomy treatment is one of the vital pulp therapies that can play a major role in the preservation of primary teeth until their natural exfoliation. The objective of this current clinical trial was to assess the clinical and radiographical success of diode and Er,Cr:YSGG lasers as a viable alternative to formocresol (FC) and sodium hypochlorite in the primary molar pulpotomies.

Materials and methods

Sixty primary molars were selected and randomly allocated to four groups. All treatment groups followed the same clinical protocol, except for the techniques used for hemostasis of the pulpotomy sites. In group A, hemostasis was achieved by applying a 1:5 dilution of FC solution, whereas in group B, 3% sodium hypochlorite was applied to achieve hemostasis. In group C, exposure to a diode laser of 940 nm was performed, whereas for group D, erbium laser irradiation with Er,Cr:YSGG laser of 2,780 nm was employed to achieve hemostasis. Radicular pulp stamps were covered with a 2 mm layer of mineral trioxide aggregate (MTA) paste. Stainless steel crowns were utilized for the final restorations of the primary teeth. The clinical and radiographic outcomes were evaluated at the six- and 12-month follow-up intervals. The investigation was registered with the ClinicalTrials.gov Protocol and Registration System (ID: NCT06002646).

Results

The overall clinical and radiographic success rates of pulpotomy were 92.3% for FC, 89% for sodium hypochlorite, 98.3% for a diode laser, and 98.7% for Er,Cr:YSGG lasers. There were no statistically significant differences among the four groups (p > 0.05).

Conclusions

Both the diode and Er,Cr:YSGG lasers showed outcomes comparable to those of FC and sodium hypochlorite. Therefore, they could be promising alternatives to primary tooth pulpotomies.

## Introduction

Primary dentition is crucial for maintaining arch length, aesthetics, mastication, speech, and preventing abnormal habits. Vital pulpotomies have been studied and suggested as a viable alternative to traditional root canal treatment (RCT) in the treatment of pulp tissue-compromised primary and permanent teeth [1-3]. There are two main categories of accepted endodontic treatment for primary teeth: vital pulp therapy (VPT) and RCT. Treating irreversible pulpal injuries and preserving pulp vitality/function are key objectives of VPT in deciduous teeth. The efficacy of VPT may be influenced by a variety of factors, including an appropriate coronal seal, sufficient blood supply, degree of inflammation, homeostasis, disinfecting the exposed site, and the antibacterial properties and biocompatibility of pulp-covering agents. Pulp vitality and, in particular, adequate vascularization, which are required for the active formation and function of odontoblasts, are essential components in the success of VPT [4].

Various techniques and materials, including formocresol (FC), sodium hypochlorite, biodentine, ferric sulfate, mineral trioxide aggregate (MTA), calcium hydroxide, and laser therapy [5-10], have been proposed for these purposes. The biological compatibility, healing capability, mutagenicity, cytotoxicity, histological response, and carcinogenic potential of these drugs or therapies vary in terms of their clinical success [11,12]. Among the many different materials, chemical agents, and techniques applied for pulp tissue management, FC remains the ‘gold standard’ for therapeutic pulpotomy in human primary teeth at a 1:5 dilution. Clinical success rates range from 70% to 97%, whereas radiographic success rates are usually lower, and overall success decreases over longer periods of follow-up [11].

The high concentration of formaldehyde in full-strength FC (19% or 190,000 ppm) makes it unsuitable for use as an effective pulpotomy medicament. This is because formaldehyde was classified as a carcinogen by the International Agency for Research on Cancer (IARC) in 2004 and again in 2011. Moreover, the United States Department of Health and Human Services (DHHS), Public Health Service, and National Toxicology Program issued the Report on Carcinogens, 12th edition, which confirmed formaldehyde's status as a recognized human carcinogen. It is crucial to explore other alternatives for medicaments with high levels of formaldehyde that interact with vascularized connective tissue in the tooth pulp of children [7,13]. Similarly, pediatric dentists largely recognize the application of sodium hypochlorite as a suitable and successful pulpotomy treatment for primary teeth [10,11]. Furthermore, several studies have reported the long-term success rate of pulpotomy with sodium hypochlorite and have recommended its application [10,11]. On the other hand, sodium hypochlorite has been shown to have local and toxic side effects, as the passage of the medication through the apical foramen can result in severe pain, ecchymosis, and swelling, as well as an unpleasant odor and taste. Sodium hypochlorite may not consistently disinfect the root canal system and can damage permanent tooth follicles, and it reacts with other irrigating solutions, such as chlorhexidine [12,14].

Due to the aforementioned concerns, limitations, and problems with the common agents applied in pulpotomy treatments, various alternative pulpotomy procedures, therapies, and materials have been suggested. In recent years, lasers have been recommended for a variety of endodontic applications, including treating dentinal hypersensitivity, pulp capping, sterilization of root canals, root canal shaping and obturation, and pulpotomy of primary and permanent teeth [3,5,6,15-17]. Recent systematic reviews have shown that lasers can enhance wound healing, are hemostatic and antimicrobial, and have cell-stimulating potential [5,8,18]. Furthermore, the laser beam has no mechanical damage to the remaining pulp tissue because it has no mechanical contact with it and only slightly increases the temperature of the pulp [8,15]. Based on these characteristics, one can claim substantial benefits of using lasers over conventional techniques for pulp therapy.

## Materials and methods

Study design

Sixty primary molar teeth in 34 patients between the ages of four and eight years, without preexisting medical conditions, were chosen from a hospital-based dental clinic. Using a convenience sampling method, 15 primary molar teeth were randomly allocated to each group based on previous trials for multiple groups and the time period available for this study [7,19]. The research was conducted in accordance with the Declaration of Helsinki and approved by the Ethics Committee of the College of Dentistry, University of Sulaimani (Registration No. 159/23 on May 3, 2023), and the trial was registered in the ClinicalTrials.gov Protocol and Registration System (ID NCT06002646).

The treatment procedures, possible discomforts, possible risks, and benefits were clarified to the parents and the patients involved, and informed consent was obtained from all the involved subjects before participation in the study. Participants were blinded to the group allocation for the pulpotomy procedure and randomly allocated to each treatment group using a simple random sampling method. After this step, both the clinical operator and patients were not blinded to the materials or devices used in the pulpotomy procedure due to the different handling methods and techniques required for each approach [1]. The study commenced on January 4, 2023. The initial follow-up period concluded on June 8, 2023, and the final 12-month follow-up was completed on April 4, 2024.

The inclusion criteria for patient participation in the trial were pediatric dental patients aged four to eight years with the primary molars exhibiting extensive carious lesions, absence of clinical and radiographic signs and symptoms of pulpal exposure or pulpal degeneration, and restorable teeth following completion of the pulpotomy procedures. The exclusion criteria were as follows: uncooperative pediatric patients, children with medically compromised disease, and the presence of clinical signs and symptoms of pulpal exposure and pulpal degeneration and physiologic root resorption of more than one-third of the primary molar roots [20].

Materials, equipment, and procedures

In this randomized, double-blind clinical trial study, pulpotomy teeth were randomly assigned to one of four groups. Control group A was treated with a 1:5 dilution of FC (Sultan Healthcare, Hackensack, NJ). Group B: 3% NaOCl (Paytekht Medical and Dental Co. Ltd., Erbil, Iraq). Experimental group C was treated with a diode laser (epic X of 940 nm; Biolase, Foothill Ranch, CA), and group D was treated with an Er,Cr:YSGG laser 2780 nm (Waterlase MD; Biolase, Foothill Ranch, CA).

**Figure 1:**
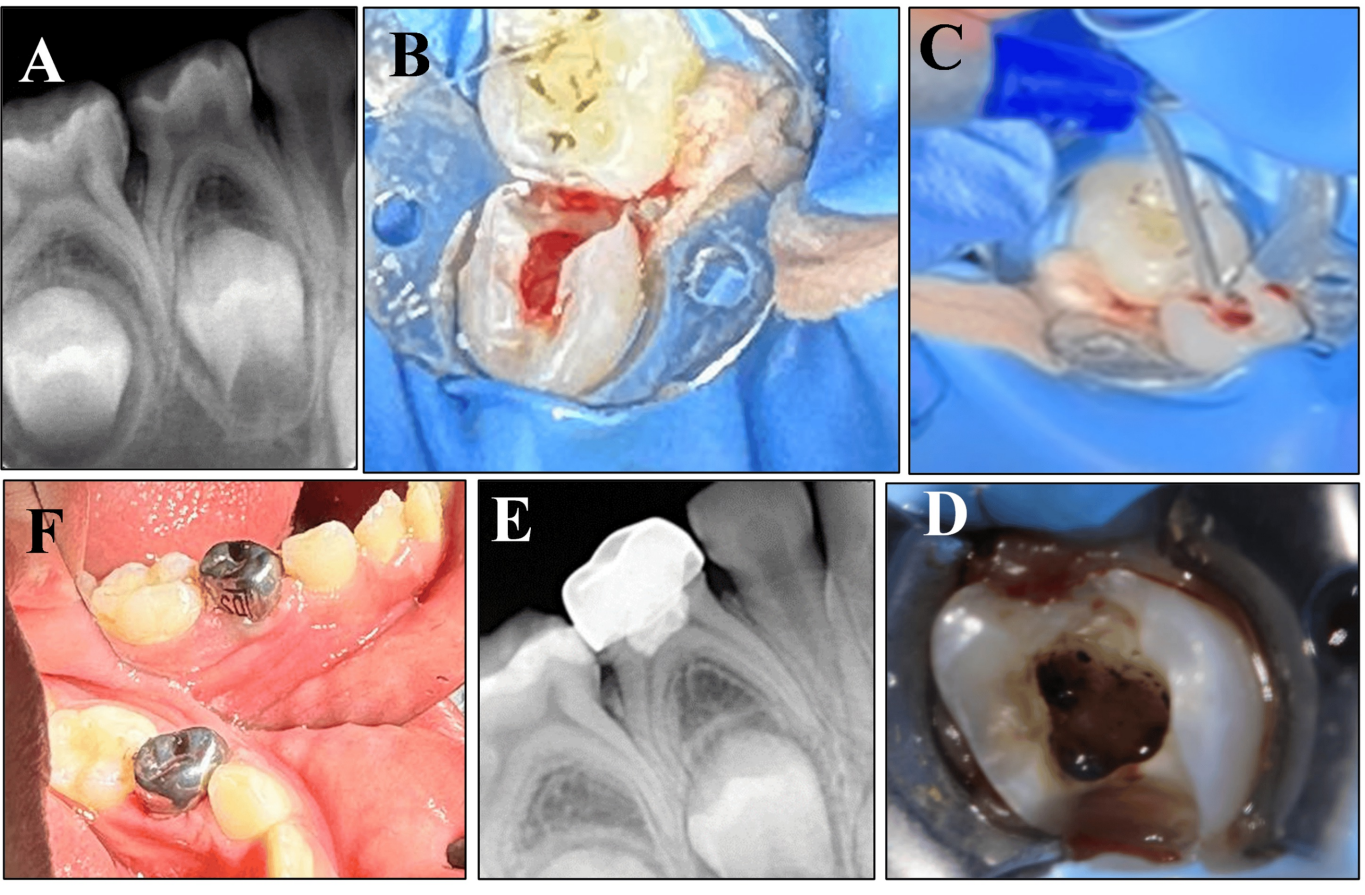
Sequence for the pulpotomy procedure using a diode laser. (A) Preoperative periapical radiograph of a first primary molar showing an extensive caries lesion on the distal side of the tooth. (B) Clinical removal of the pulp chamber roof and subsequent pulp exposure and pulp amputation. (C) and (D) Hemostasis of the pulpotomy sites using the diode laser. (E) Periapical radiograph showing the final restoration of the tooth. (F) Clinical photograph of the restored first primary molar.

**Figure 2:**
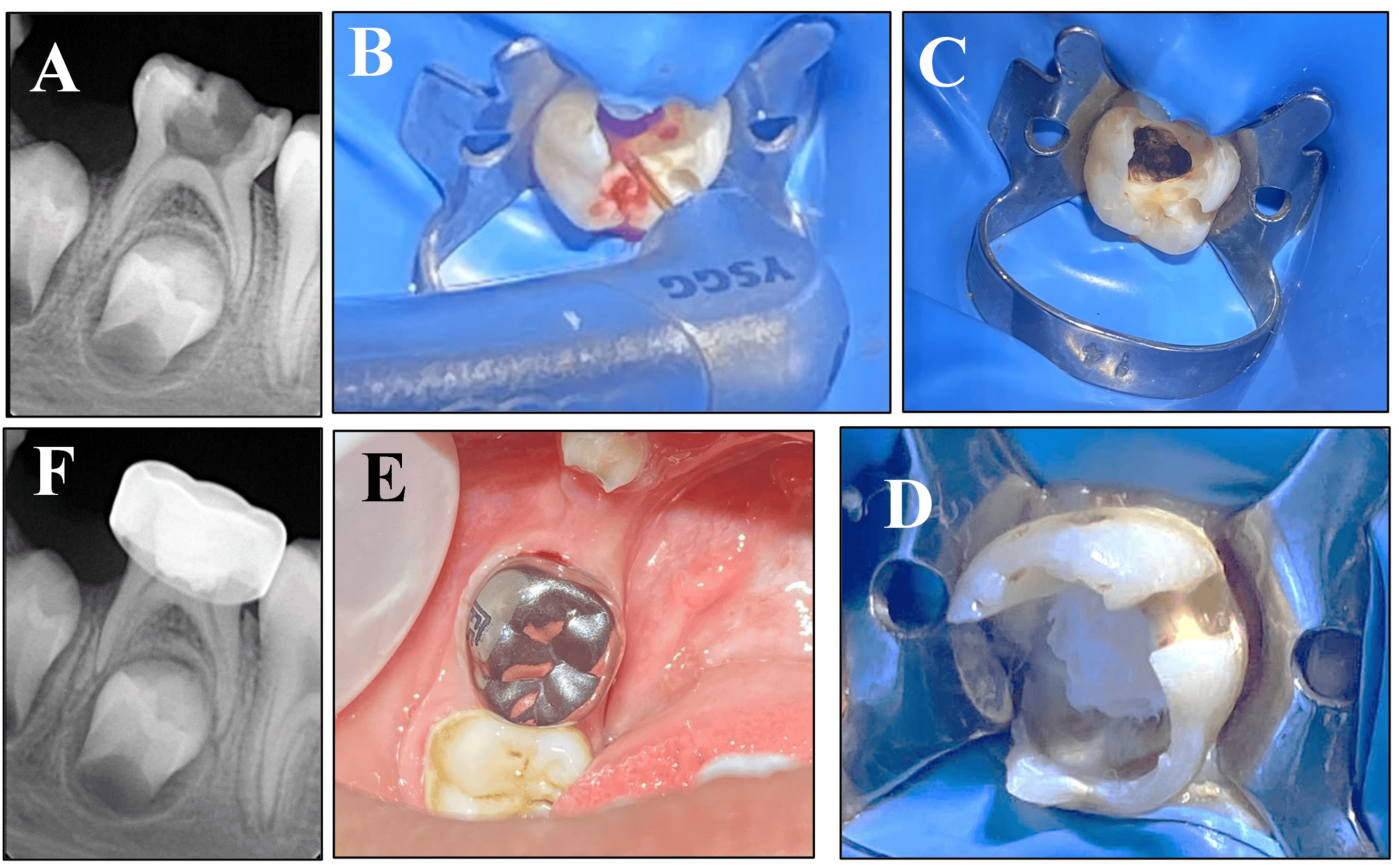
Sequence for the pulpotomy procedure using the Er,Cr:YSGG laser. (A) Preoperative periapical radiograph of a second primary molar showing an extensive caries lesion on the occlusal surface of the tooth. (B) Hemostasis of the pulpotomy sites using the Er,Cr:YSGG laser after the removal of the pulp chamber and pulp amputation. (C) Pulp stamps after laser application. (D) Application of a layer of the mineral trioxide aggregate (MTA) material to cover the pulpotomy site. (E) and (F) Clinical photograph and periapical radiograph showing the final restoration of the tooth.

The pulpotomy process involved the following steps: After applying local anesthesia and isolating the tooth with a rubber dam, the caries were removed, and the pulp chamber was unroofed using a high-speed bur. The contaminated pulp tissue was meticulously removed using a sterile sharp spoon excavator, followed by a thorough irrigation with sterile saline solution. In control group A, after amputation of the pulp and initial control of bleeding with damp cotton, complete hemostasis was achieved by applying a 1:5 dilution FC cotton pallet for five min. In group B, a sterile cotton pellet was moistened with 3% sodium hypochlorite and placed in the pulp chamber for five minutes to achieve hemostasis. Group C achieved complete hemostasis via irradiation of the area to a diode laser (940 nm) operating at a power of 2 W in continuous wave mode (CW). To achieve full hemostasis, laser energy was applied to the canal orifice using a 300-um optical fiber in contact mode. It was done for one second at each orifice (Figure 1). In group D, irradiation with Er,Cr:YSGG laser 2780 nm at a power of 1.5 W, frequency of 50 Hz, S mode (soft tissue mode), 20% air, and no water with a gold handpiece, and tip-type MZ6 was applied for 10 seconds until a fixed char layer formed over the pulpal tissue of the canal orifices (Figure 2). During laser application, patients, operators, and assistants used protective eye shields, according to the safety measures of the device user manual.

Around a 2 mm layer of MTA (NeoPUTTY; Avalon Biomed, Houston, TX) was used to cover the pulpal stumps in all groups. A stainless steel crown was used to restore the treated teeth (3M ESPE™ Stainless Steel Crowns). Clinical follow-up was performed every six months after treatment. All pulpotomy procedures were performed by the same investigator. At each follow-up visit, the treated teeth and their radiographs were evaluated separately by the principal investigator and the second examiner dentist.

Criteria for success of the pulpotomy treatment

Clinical success was achieved in the absence of pain, tenderness, pathological mobility, and soft tissue pathology (fistula or swelling). Radiographic success was achieved without internal or external root resorption, as well as periapical or furcal radiolucency. When one or more of the failure criteria indicators were present, the treatment was deemed to have failed [5,18].

Statistical analysis

Statistical Product and Service Solutions (SPSS, version 25.0; IBM SPSS Statistics for Windows, Armonk, NY) categorizes and summarizes the data using percentages and frequencies used for all statistical analyses. The chi-square and Fisher's exact test were used at each follow-up evaluation to report differences in the success and failure rates among the four groups. Statistical significance was set at p < 0.05. Intra- and inter-examiner agreement of radiographic assessments was evaluated using the kappa test.

## Results

**Figure 3:**
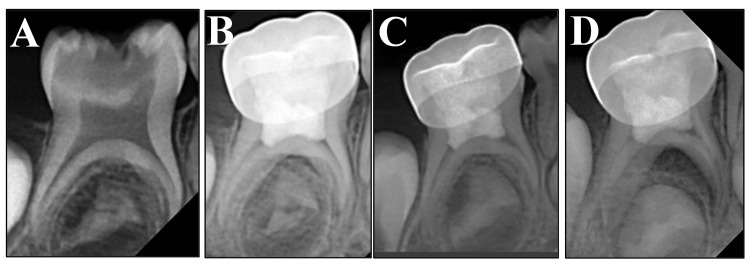
Periapical radiographs of a successful case. (A) Periapical radiograph of a second primary molar showing extensive occlusal caries. (B) Immediate postoperative radiograph after the pulpotomy procedure. (C) and (D) 6- and 12-month follow-up periapical radiographs showing no bone or root resorption. Note the continued normal development of the underlying premolar successor tooth crown.

**Figure 4:**
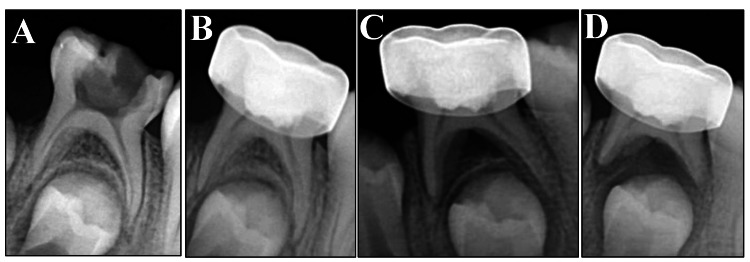
Periapical radiographs of a failed case. (A) Periapical radiograph of a primary molar showing extensive occlusal caries. (B) Immediate postoperative radiograph after the pulpotomy procedure. (C) Periapical bone and root resorption at the six-month follow-up. (D) 12-month follow-up periapical radiograph showing continued bone and root resorption involving all the primary tooth roots and the bifurcation area.

A total of 34 children (19 females and 15 males) aged between four and eight years (mean age = 6±1.02 years) participated in this study. Among the 60 individual primary molars evaluated in this study, 56 were clinically successful for more than 12 months. Four clinical and radiographical failures were observed within the first six months of the study. The failures presented with either pain and/or tenderness and soft tissue pathology, with three patients presenting with periapical and inter-radicular radiolucency (Table 1). Figures 3-4 show the sequence of periapical radiographs of a successful and a failed patient at the time of the study. There were no observable differences in the clinical or radiographic outcomes among the four groups at the six-month (p > 0.05) or 12-month (p > 0.05) follow-up. The kappa value for intraexaminer reliability was 0.867, and that for interexaminer reliability was 0.8.

**Table 1:** The number of clinical and radiographical failures that occurred within 12 months.

Clinical Criteria	FC	NaOCl	Diode Laser	Er,Cr:YSGG Laser
External root resorption	7	5	3	1
Internal root resorption	0	0	0	1
Periapical radiolucency	1	1	0	0
inter-radicular radiolucency	1	1	0	0
Pain	1	2	0	0
Tenderness	1	2	0	0
Swelling	0	2	0	0
Fistula	1	3	0	0
Pathological mobility	0	0	0	0
The clinically symptom-free radiographic failures after 12 months were 12 cases				

At the six-month follow-up, the clinical success rates were 96% for the FC, 88% for the sodium hypochlorite, and 100% for both the diode and Er,Cr:YSGG lasers (Table 2). The outcomes of the clinical assessments remained consistent with the conclusion of the 12-month follow-up period. On the other hand, the radiographic success rates during the six-month and 12-month follow-ups were 92%-85% for FC, 92%-88% for sodium hypochlorite, 98%-95% for the diode laser, and 98%-96.7% for the erbium laser (Table 2). There were no significant differences in the clinical or radiographic success rates among the four groups at the different follow-up intervals.

**Table 2:** Clinical and radiographic success rates for each treatment group at both the six-month and 12-month follow-up periods.

Treatment Groups	Clinical Success Rate (6 Months)	Clinical Success Rate (12 Months)	Radiographic Success Rate (6 Months)	Radiographic Success Rate (12 Months)
Formocresol (FC)	96%	96%	92%	85%
Sodium Hypochlorite (NaOCl)	88%	88%	92%	88%
Diode Laser	100%	100%	98%	95%
Er,Cr:YSGG Laser	100%	100%	98%	96.7%

In the group treated with FC, at the six-month mark, the clinical success rate was 96%, indicating that 14 out of 15 patients exhibited positive outcomes without clinical failure. This rate remained consistent at the 12-month follow-up. However, the radiographic success rate decreased slightly, from 92% at six months to 85% at 12 months. Notably, seven patients in this group showed evidence of external root resorption, and six of them did not experience clinical symptoms (Table 1). Additionally, one patient discontinued the study because of pain, tenderness, and pathological radiolucency (Table 3).

**Table 3:** Number of cases discontinued from each treatment group at the end of the study.

Treatment Groups	Number of Cases Discontinued From the Study	
	Yes	No
Formocresol (FC)	1	14
Sodium Hypochlorite (NaOCl)	3	12
Diode Laser	0	15
Erbium Laser (Er,Cr:YSGG)	0	15

Similarly, in the sodium hypochlorite group, the clinical success rate was 88% at both six and 12 months, with 12 of 15 cases demonstrating positive outcomes. The radiographic success rate remained consistent at 92% at six months and 88% at 12 months. Three patients in this group exhibited external root resorption without clinical manifestations (Table 1). In comparison, three patients discontinued the study due to various clinical presentations, including pain, tenderness, swelling, fistula, and pathological periapical radiolucency (Table 3).

In the diode laser group, all 15 cases achieved clinical success at both six and 12 months, yielding a 100% success rate. The radiographic success rate was 98% at six months and decreased slightly to 95% at 12 months. Three patients in this group presented with external root resorption not accompanied by any clinical symptoms. In the Er,Cr:YSGG laser group, notable findings included one case exhibiting external root resorption and another showing internal root resorption without any clinical signs or symptoms. Radiographical evaluations demonstrated a success rate of 98% at the six-month follow-up, which marginally decreased to 96.7% at the end of the 12-month follow-up period (Table 2).

Notably, the overall clinical and radiographical success rates at the end of the study period were 96% and 88.5% for the FC, 88% and 90% for the sodium hypochlorite, 100% and 96.5% for the diode laser, and 100% and 97.3% for the Er,Cr:YSGG laser, respectively (Table 4).

**Table 4:** The combined success rates for both clinical and radiographic evaluations across both the six-month and 12-month follow-up periods for each treatment group.

Treatment Groups	Overall Clinical Success Rate	Overall Radiographic Success Rate
Formocresol (FC)	96%	88.5%
Sodium Hypochlorite (NaOCl)	88%	90%
Diode Laser	100%	96.5%
Erbium Laser (Er,Cr:YSGG)	100%	97.3%

## Discussion

Compared with permanent teeth, primary teeth are more vulnerable to early pulp involvement because they have thinner enamel and dentin, larger pulp chambers, and wider dentinal tubules [15]. When pulp inflammation remains confined to the coronal part of the pulp, pulpotomy is the preferred treatment option [21]. Pulpotomy is 'a dental procedure performed on a tooth with a deep carious lesion near the pulp, involving the removal of the coronal pulp to maintain the vitality of the radicular pulpal tissue' [12,21].

Vital pulpotomy has long been a topic of debate. This randomized clinical study is considered one of a limited number of clinical trials in which lasers were employed for pulpotomy in primary molars in children, with only a few studies involving diode lasers [7,9,22], an animal study by Toomarian et al. using Er,Cr:YSGG [23] and a nine-month follow-up study by Ramanandvignesh et al. combining Er,Cr:YSGG lasers with MTA and biodentine in primary teeth [15]. Owing to the decontamination property of the Er,Cr:YSGG laser, bactericidal action can be achieved; thus, Er,Cr:YSGG can be effectively used for pulpotomy procedures. In an animal study, Toomarian et al. [23] investigated whether Er,Cr:YSGG laser pulpotomy is an acceptable alternative for FC and determined that the Er,Cr:YSGG laser resulted in 'superior preservation of the odontoblastic layer, reduced odontoclast count, and decreased occurrences of hemorrhage, inflammation, internal resorption, tissue necrosis, vascularization, and abscess development compared to the formocresol' [23]. The current clinical trial was conducted to evaluate the success rate of the laser pulpotomy technique in human primary molars clinically and radiographically and to compare it with that of the FC and sodium hypochlorite pulpotomy techniques.

FC was used as the standard control technique for pulpotomy due to its established long-term clinical efficacy despite concerns regarding adverse effects [13]. Sodium hypochlorite (NaOCl) was explored as an alternative option. Considering its recent positive outcomes in diverse clinical applications, NaOCl was also explored as a viable alternative to FC [10,24].

The overall success rates at six months were 94% for FC; 90% for NaOCl; 99% for the diode; and 92.3%, 89%, 98.3%, and 98.7% for the Er,Cr:YSGG lasers at the end of 12 months. The most common clinical failures in the present study were pain, tenderness to percussion, and fistula. Radiographic failure was attributed to interradicular radiolucency and external root resorption, with one of the failures in group A attributed to loss of full coverage restoration, and the remaining three failures in group B were children who presented with symptoms of pain, tenderness, and/or soft tissue pathologies. Three patients in this group exhibited external root resorption without clinical manifestations. In comparison, three patients discontinued the study due to various clinical presentations, including pain, tenderness, swelling, fistula, and periapical radiolucency. These results were consistent with the findings of a recent systematic review and meta-analysis by Coll et al. in 2017, indicating a reduced clinical and radiographic success rate associated with sodium hypochlorite compared with the established standard of FC [24]. Poor tooth selection, failure of the pulpotomy medication, technical error, and failure of the restoration are all possible causes of these failures [12].

No statistically significant differences were found among the four groups, and these outcomes correspond with studies carried out on the pulpotomy treatment of primary molars that suggested that MTA, laser, or biodentine demonstrated efficacy comparable to that of FC and can be regarded as viable substitutes [7,9,15,19]. Furthermore, pulpotomies performed with different lasers have been reported to be either superior or equivalent to FC pulpotomies [8,18].

The high rate of radiographic failure regarding external root resorption in this study suggests age-related natural resorption of the root rather than being pathological because these failures are not correlated with clinical signs and symptoms [25]. The study results are consistent with those of previous pulp therapy studies in that the observed radiographic success rate was lower than the clinical success rate [26]. This is partly attributable to radiographic failure not consistently correlating with the signs or symptoms of clinical failure, such as pathologic mobility, pain, or soft tissue pathology. For example, a tooth with radiographic signs of internal root resorption in group D, although considered a radiographic failure, did not present with any clinical signs of failure until the end of the study period.

Our study aligns with previous research suggesting the viability of laser pulpotomy as a substitute for conventional techniques. Despite the variability in reported outcomes across different studies, our findings contribute to the overall understanding of pulpotomy efficacy and support the consideration of lasers as a promising option for vital pulp therapy in primary molars. Further research is warranted to elucidate the long-term outcomes and potential benefits of laser pulpotomy, particularly in a larger and more diverse patient population. Comparative studies exploring various laser types and parameters could provide valuable insights into optimizing treatment protocols to improve clinical outcomes. Overall, our study underscores the importance of continued exploration and innovation in vital pulp therapy to improve dental care outcomes in pediatric patients. Laser therapy is a viable substitute for FC in pulpotomy procedures for the treatment of the pulp of primary teeth. Further research with extended periods of observation is necessary to provide a definitive set of guidelines for the treatment of deep caries lesions in primary teeth using pulpotomy procedures.

Due to the limited resources and time available for this trial, limitations of this study include the limited no of selected materials and techniques that were tested against each other in the pulpotomy treatment plan and the restricted 12-month follow-up time frame. There are many other types of lasers and medicaments that could be tested on a larger number of patients for longer clinical follow-ups, 24 months or 36 months. This study also does not include permanent teeth, and pulpotomy techniques can be very useful in saving pulp tissue-compromised young permanent teeth due to extensive caries lesions or traumatic dental injuries.

## Conclusions

In conclusion, the present clinical trial showed high clinical and radiographic success rates at the end of 12 months. Through meticulous examination of the outcomes over the 12-month period, we observed comparable success rates among all the tested treatment groups. Laser pulpotomy, specifically using diodes and Er,Cr:YSGG lasers, has demonstrated favorable results akin to, if not better than, conventional methods. However, considering certain factors, such as the cost and clinical experience required for lasers, the use of lasers is somewhat restricted.
